# Untargeted GC-MS-Based Metabolomics for Early Detection of Colorectal Cancer

**DOI:** 10.3389/fonc.2021.729512

**Published:** 2021-11-04

**Authors:** Guoxue Zhu, Yi Wang, Wang Wang, Fang Shang, Bin Pei, Yang Zhao, Desong Kong, Zhimin Fan

**Affiliations:** ^1^Department of Neurology, Nanjing Hospital of Chinese Medicine Affiliated to Nanjing University of Chinese Medicine, Nanjing University of Chinese Medicine, Nanjing, China; ^2^Department of Anorectal Medicine, Nanjing Hospital of Chinese Medicine Affiliated to Nanjing University of Chinese Medicine, Nanjing University of Chinese Medicine, Nanjing, China; ^3^School of Medicine & Holistic Integrative Medicine, Nanjing University of Chinese Medicine, Nanjing, China; ^4^Chinese Medicine Modernization and Big Data Research Center, Nanjing Hospital of Chinese Medicine Affiliated to Nanjing University of Chinese Medicine, Nanjing University of Chinese Medicine, Nanjing, China

**Keywords:** colorectal cancer, metabolomics, GC-MS, cancer tissue and paracarcinoma tissue, preoperative and postoperative serum

## Abstract

**Background:**

Colorectal cancer (CRC) is one of the most common malignant gastrointestinal cancers in the world with a 5-year survival rate of approximately 68%. Although researchers accumulated many scientific studies, its pathogenesis remains unclear yet. Detecting and removing these malignant polyps promptly is the most effective method in CRC prevention. Therefore, the analysis and disposal of malignant polyps is conducive to preventing CRC.

**Methods:**

In the study, metabolic profiling as well as diagnostic biomarkers for CRC was investigated using untargeted GC-MS-based metabolomics methods to explore the intervention approaches. In order to better characterize the variations of tissue and serum metabolic profiles, orthogonal partial least-square discriminant analysis was carried out to further identify significant features. The key differences in t_R_–m/z pairs were screened by the S-plot and VIP value from OPLS-DA. Identified potential biomarkers were leading in the KEGG in finding interactions, which show the relationships among these signal pathways.

**Results:**

Finally, 17 tissue and 13 serum candidate ions were selected based on their corresponding retention time, p-value, m/z, and VIP value. Simultaneously, the most influential pathways contributing to CRC were inositol phosphate metabolism, primary bile acid biosynthesis, phosphatidylinositol signaling system, and linoleic acid metabolism.

**Conclusions:**

The preliminary results suggest that the GC-MS-based method coupled with the pattern recognition method and understanding these cancer-specific alterations could make it possible to detect CRC early and aid in the development of additional treatments for the disease, leading to improvements in CRC patients’ quality of life.

## Introduction

Colorectal cancer (CRC) is a severe health problem and ranks as the third leading cause of tumor-causing death in Europe and the USA (1). And while there are many factors, including the environment, alcohol consumption and smoking are believed to increase the incidence of CRC (1). The pathogenetic progression of colorectal cancer is closely related to polyps (2). Most of the CRCs arise from adenomas, beginning as polyps on the inner wall of the colon or rectum, and subsequently intravasating into lymph vessels or blood vessels, increasing the chance of disseminating to other organs (3). Detecting and removing these malignant polyps promptly is the most effective method at present in CRC prevention. Therefore, the analysis and disposal of malignant polyps is conducive to preventing CRCs.

Currently, there are ways to relatively detect CRCs, such as colonoscopy (4), computed tomography colonography (5), fecal occult blood test (6), and multitarget stool DNA testing (7). However, it has some disadvantages, bleeding risk, inconvenience, no cost-effectiveness, and lower sensitivity and specificity. Unfortunately, most CRC patients are diagnosed when they are in the late stages of the disease with metastasis, making it harder to achieve complete remission. The development of reliable and predictive biomarkers would be a critical tool to identifying individuals with evolving CRC or presence of early disease. However, there is still no tissue or serum biomarker that can be utilized for contented CRC diagnosis. It is urgent to find new screening methods with sensitive, specific, convenient, and non-invasive characters for the early diagnosis of CRC.

Metabolomics is a high-throughput tool useful for exploring metabolites by detecting small-molecule metabolites using mass spectrometry (<1,800 Da). For this reason, small variations in the body can indicate early biological changes to the host due to perturbations in metabolic pathways. Therefore, the metabolome could be regarded as the amplified output of a biological system (8, 9). Monitoring fluctuations of certain metabolite levels in body fluids has become an important way to detect early stages in CRC. High-throughput analytical technologies for metabolomics, such as nuclear magnetic resonance (NMR) and mass spectrometry (MS), seem imperative in an untargeted type (10). Recent technological advances allow for the establishment of systematic, holistic methods to relatively short analysis times. In addition, chromatographic methods, such as LC and GC, offer a significant advantage to the MS detection allowing the identification of metabolites based on their chemical properties. Compared to conventional liquid chromatography–tandem mass spectrometry (LC-MS) instruments, gas chromatography–tandem mass spectrometry (GC-MS) detection has gained popularity with its higher chromatographic resolution, reproducibility, and robustness which allowed the establishment of a comprehensive database of identified peaks (11).

**Figure 1:**
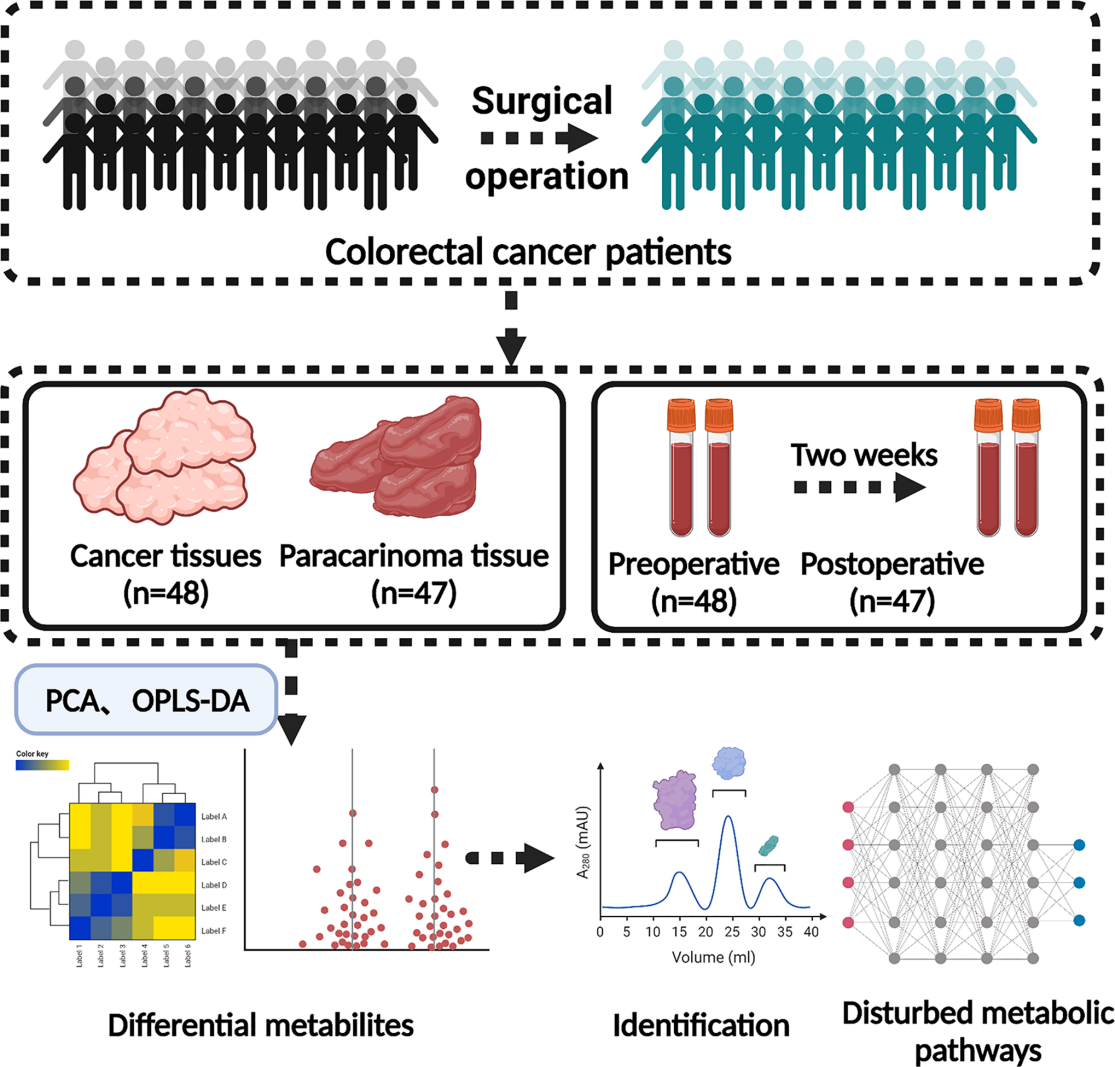
The schematic diagram of the experimental design.

Herein, GC-MS-based tissue metabolomics was applied for analyzing the difference between cancer tissue and paracarcinoma tissue in CRC patients. Simultaneously, GC-MS-based serum metabolomics between preoperative and postoperative (2 weeks) CRC patients is a significant auxiliary for the sake of explicating metabolic pathway transformation and establishing a panel of biomarkers, which would be of diagnostic significance to CRC (**Figure 1**).

## Material and Methods

### Study Population and Sample Collection

All the experiments of human specimens were in accordance with the ethical code and recommendation issued by the Ethics Committee of Human Experimentation and Chinese Animal Community and with the Helsinki Declaration (approval number: KY2020081). The tissue and serum samples were collected from 48 patients diagnosed with CRC undergoing colorectal resection in Nanjing Hospital of Chinese Medicine Affiliated to Nanjing University of Chinese Medicine. Clinical information, including patient samples, tumor size, and clinical staging, and other information on these CRC patients are shown in **Table 1** and **Supplemental Information Table S1**. The patients we collected came from various localities and owned various living environments and personal habits. Histopathologic examination and immunohistochemistry were utilized to confirm the diagnosis of CRC. Concurrently, the inclusion criteria of the CRC patients in this research included the following: (1) age between 35 and 85 years; (2) conforming to the “Diagnosis, management, and treatment of colorectal cancer” (2015) standard; (3) clear and definite preoperative diagnosis; (4) patients agree to become involved in clinical trials. Exclusion criteria were as follows: (1) gestational period and suckling period of female; (2) malignant hematopathy, including all types of leukemia and anemia; (3) patients with significant metabolic abnormalities; (4) patients with primary tumors in other parts of the body; (5) patients with allergic constitution; (6) use of specific drugs during the last 3 weeks, such as antibiotics, hormones, and non-steroid anti-inflammatory drugs.

**Table 1:** Clinical features of colorectal cancer patients enrolled in this study.

Characteristics	Number
Gender (male/female)	28/20
Age (mean ± SD) [min, max]	65 ± 12 [36, 85]
Median age among male	67
Median age among female	67
Blood glucose (mmol/L) (mean ± SD) [min, max]	5.07 ± 0.63 [3.90, 6.80]
Body mass index (kg/m^2^) (mean ± SD) [min, max]	22.95 ± 3.20 [17.19, 30.39]
Tumor sites	
Colorectal (number)	21
Sigmoid colon (number)	10
Descending colon (number)	2
Ascending colon (number)	15
Pathologic subtypes	
Adenocarcinoma (number)	46
Mucoid carcinoma	2
Tumor size (cm) [min, max]	2.5 × 2 × 0.8, 11 × 7 × 3
Differentiation	
Low	6
Medium-low	10
Medium	31
High	1
Infiltration	
Adventitia	11
Deep myometrial	4
Serosal	26
Submucosal	6
Peripheral adipose	1
Tumor stage	
I	7
II	19
III	21
IV	1

### Sample Preparations

The tissue (cancer tissue and paracarcinoma tissue) and serum (preoperative and postoperative of 2 weeks) samples were screened from 48 CRC volunteers taken from Nanjing Hospital of Chinese Medicine Affiliated to Nanjing University of Chinese Medicine. After resection of CRC patients, tumor tissue was collected in the central area of solid tumor immediately, and paracancerous tissue was collected in the area about 5 cm away from the solid tumor. A 100-mg sample was taken and then mixed with 10 μl methanol solution that consists of internal standard solution of heptadecanoic acid (1 mg/ml) into 1.5-ml EP tubes, extracted with 0.4 ml extraction liquid (V (methanol): V (chloroform) = 3:1), and vortexed for 30 s at room temperature. Following centrifugation at 13,000 rpm for 10 min at 4°C, the resulting 300-μl supernatant was transferred into a sample vial for vacuum drying at room temperature. The residue was redissolved in 40 μl of a methoxyamine solution (15 mg/ml in pyridine) and vortexed for 1 min. An oximation reaction was performed at 37°C for 1.5 h. An 80-μl aliquot of BSTFA containing 1% trimethylchlorosilane (TMCS) was then added to the solution, and vortex oscillation for 30 s followed. The obtained samples were then kept at 70˚C in an oven for 1 h before weighing, and a 40-μl aliquot of acetonitrile was added. Samples were then centrifuged at 13,200 rpm for 10 min at 4°C. The supernatant was transferred to an autosampler vial for GC-MS analysis.

Ten-microliter internal standard solutions (heptadecanoic acid in methanol, 1 mg/ml) and extraction liquid (V (methanol): V (chloroform) = 3:1, 600 µl) were added to serum samples. The mixture was vortexed for 30 s, and the mixture was stored at 37˚C for 10 min. The resulting 600 -μl supernatant was transferred into a sample vial for vacuum drying at room temperature. The remaining methods are as aforementioned.

### Gas Chromatography–Tandem Mass Spectrometry Conditions

The samples were analyzed using an Agilent 7890 chromatograph coupled with a 5977B MS system (Agilent Technologies, USA) and EI Source. A DB-5 ms capillary column which was coated with 95% dimethyl/5% diphenyl polysiloxane (30 m × 0.25 mm inner diameter i.d., 0.25-µm film thickness, CA, USA) was utilized in the separation system. The temperature procedure of the column was established as follows: initially, the GC oven temperature was maintained at 60°C for 1 min, then the temperature was raised to 325°C at a rate of 10°C/min and then maintained at 325°C for 10 min. The temperature of the inlet and ion source was maintained at 250°C, respectively. The injection volume was 1 μl. Helium was utilized at a constant flow rate of 0.87 ml/min as the carrier gas. The MS system was operated with electron impact ionization at 70 eV and a scanning range of m/z 50–650 (full-scan mode).

### Data Processing and Statistical Analysis

QC samples were analyzed five times at the beginning of the run and injected once after every 10 injections of the random sequenced samples. The raw data obtained from the GC-MS run were transformed to the mzData format using MassHunter Workstation Software (Version B.06.00, Agilent Technologies). Data pretreatments including non-linear retention time alignment, peak discrimination, filtering, alignment, matching, and identification were done using the XCMS Online platform (https://xcmsonline.scripps.edu). The obtained three-dimensional data which were generated from XCMS were conducted by principal component analysis (PCA) and orthogonal partial least square discriminant analysis (OPLS-DA) using SIMCA-P software (version 14.0, Umetrics, Sweden). The t-test was utilized to compare the significant difference between cancer tissue and paracarcinoma tissue for parametric variables. For each statistical analysis, a p-value less than 0.05 was considered as significant. The metabolites with variable importance on the projection (VIP) value greater than 1.0 and p values less than 0.05 were set for differential metabolites.

### Biomarker Identification and Pathway Enrichment Analysis

Only the variables according with the criteria of “p-value <0.05 in ANOVA and VIP values ≥1.0” were screened as potential biomarkers, and then research for the molecular mechanism was continued. The elemental formula and fragmentation patterns were obtained using MassHunter Workstation Software. Simultaneously, differential metabolites were tentatively identified by library search (NIST for example), Human Metabolome Database (http://www.hmdb.ca/), METLIN (https://metlin.scripps.edu/), and MassBank (http://www.massbank.jp/). The pathway analysis for metabolites was performed by KEGG using MetaboAnalyst 5.0 (http://www.metaboanalyst.ca/).

## Results

### Confirmatory Studies by Histopathologic Examination and Immunohistochemistry

The CRC samples utilized for immunohistochemistry staining were formalin-fixed, paraffin-embedded tissues and included tissues of the CRC tumor from each patient. Fifty cases of CRC were collected and spotted in duplicate. Diagnostic paraffin blocks were selected on the basis of the availability of suitable formalin-fixed paraffin-embedded tissue. A histological confirmation of CRC was achieved in all cases by a central review using standard tissue sections, and most of the tumor-rich areas were marked in the paraffin blocks. Finally, the CRC was confirmed by hematoxylin and eosin (H&E) staining (**Figure S1**).

### Result of Multivariate Statistical Analysis

**Figure 2:**
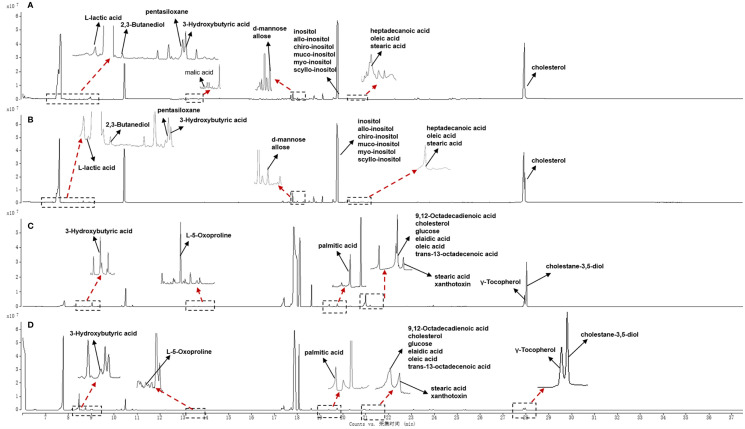
Total ion chromatogram of cancer tissue **(A)** and paracarcinoma tissue **(B)** and preoperative **(C)** and postoperative **(D)** serum of 2 weeks in positive-ion mode.

**Figure 3:**
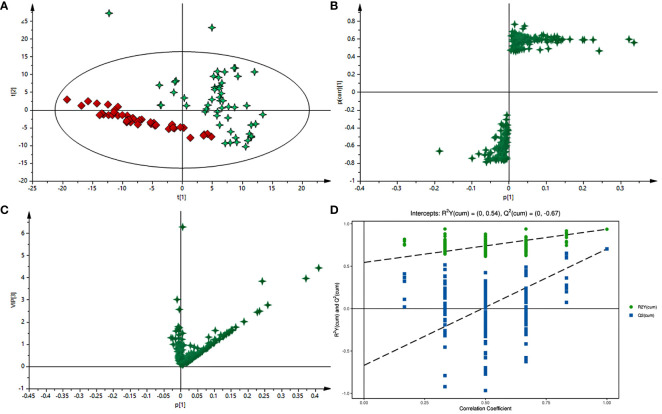
**(A)** PCA score plot of cancer tissue and paracarcinoma tissue samples in positive-ion mode with the statistical parameters (R^2^X = 0.964, Q^2^ = 0.897). **(B)** S-plot of OPLS-DA with the statistical parameters in positive-ion mode (R^2^X = 0.940, R^2^Y = 0.756, Q^2^ = 0.743). **(C)** VIP value plot between cancer tissue and paracarcinoma tissue samples in positive-ion mode. **(D)** Validation plot of the cancer tissue and paracarcinoma tissue samples in positive-ion mode obtained from 200 permutation tests.

**Figure 4:**
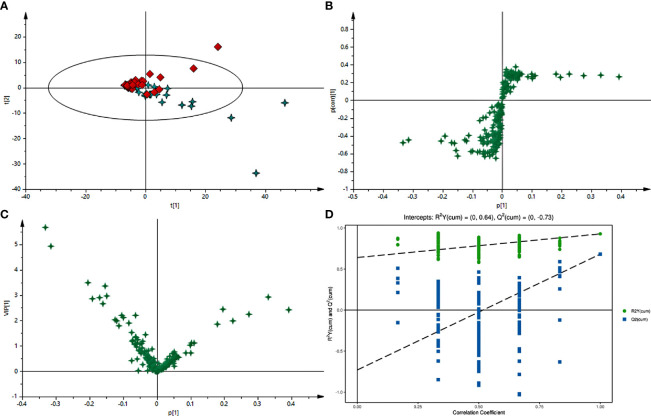
**(A)** PCA score plot of preoperative and postoperative serum of 2-week samples in positive-ion mode with the statistical parameters (R^2^X = 0.965, Q^2^ = 0.823). **(B)** S-plot of OPLS-DA with the statistical parameters in positive-ion mode (R^2^X = 0.939, R^2^Y = 0.639, Q2 = 0.844). **(C)** VIP value plot between preoperative and postoperative serum of 2-week samples in positive-ion mode. **(D)** Validation plot of the preoperative and postoperative serum of 2-week samples in positive-ion mode obtained from 200 permutation tests.

Typical GC-MS base peak intensity (BPI) chromatograms of tissue samples from the cancer and paracarcinoma and serum samples from the preoperative and postoperative CRC were obtained (**Figure 2**). Firstly, we specifically compared the tissue and serum samples for the sake of unambiguous classification. Supervised PCA was conducted on the samples to visualize general clustering, trends, or outliers among the observations. As shown in **Figure 3A**, the clustering significantly differed between the cancer and paracarcinoma tissues, implying the chemical composition in the significant diversity of tissues between two groups. Simultaneously, the PCA analysis results for serum are shown in **Figure 4A**. In order to better characterize variations of tissue and serum metabolic profiles, orthogonal partial least-square discriminant analysis was carried out to further identify significantly features/ions. The key differences in t_R_–m/z pairs were screened by the S-plot and VIP value (**Figures 3C** and **4C**) from OPLS-DA, respectively. In the OPLS-DA S-plot (**Figures 3B** and **4B**), each variation point represents an t_R_–m/z pair; the X-axis represents variable contribution which has a further distance from the origin point, the greater the contribution to the separation of groups; the Y-axis represents variable confidence which has a further distance from the origin point, the higher the confidence level of the t_R_–m/z pairs to the separation of groups (12). The OPLS-DA model was further validated by cross-validation and permutation test (**Figures 3D** and **4D**). Furthermore, the ions with variable importance in the projection (VIP) values >1.0 and p-value <0.05 in ANOVA that at the corner of S-plot were the variables dedicating most to the differences.

### Identification of Potential Biomarkers and Pathway Analysis of CRC

**Figure 5:**
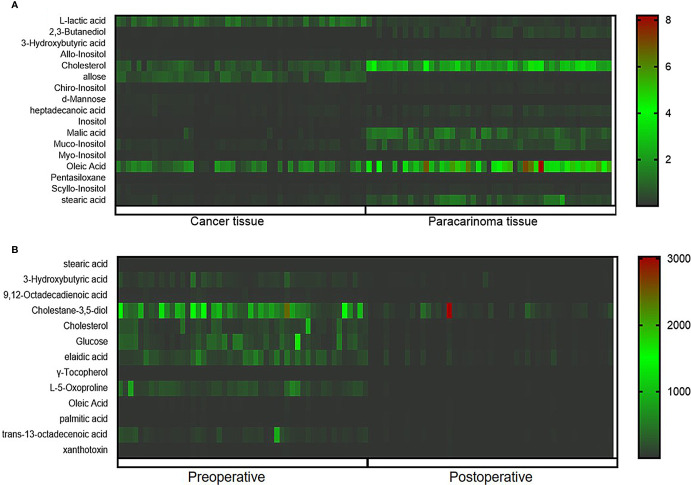
Heatmap of differential metabolites between cancer tissue and paracarcinoma tissue samples **(A)** and preoperative and postoperative serum of 2-week samples **(B)**.

The ions with VIP ≥1.0 and p < 0.05 obtained from the S-plot were considered as the candidate putative biomarkers for NIST, METLIN, MetaboAnalyst, and Human Metabolome Database identification. Finally, 17 tissue and 13 serum candidate ions were selected and their corresponding retention time, p-value, m/z, and VIP value are summarized in **Table 2**, and the heat maps of the tissue and serum samples were conducted (**Figure 5**). Simultaneously, the receiver-operating characteristic (ROC) curve was utilized to evaluate the potential biomarkers (**Figure S2**). The results show that the trends of stearic acid and cholesterol are consistent, which were the most promising biomarkers.

**Table 2:** The identification of potential biomarkers in tissue level based on GC-MS.

Differential metabolites	m/z	RT	VIP	Fold change	p
**Cancer tissue and paracarcinoma tissue**					
L-Lactic acid	318.0358	7.31	1.15	2.87	1.15E-10
2,3-Butanediol	116.0217	7.78	1.16	0.19	9.97E-06
3-Hydroxybutyric acid	373.0875	8.94	1.26	3.07	2.43E-16
allo-Inositol	405.1945	19.73	1.07	0.49	9.11E-09
Cholesterol	75.0181	27.94	1.06	0.39	1.12E-08
Allose	97.0683	17.98	1.21	4.02	7.61E-15
chiro-Inositol	381.1673	19.73	1.05	0.51	5.93E-08
d-Mannose	138.0889	17.98	1.23	5.89	6.43E-16
Heptadecanoic acid	146.0376	20.24	1.01	0.40	3.63E-08
Inositol	391.1874	19.73	1.12	0.48	1.25E-08
Malic acid	233.0896	13.2	1.05	0.09	1.40E-09
Muco-inositol	407.1886	19.73	1.01	0.53	6.86E-08
myo-Inositol	164.0926	19.76	1.24	9.40	7.65E-21
Oleic acid	117.0078	20.24	1.02	0.39	6.86E-08
Pentasiloxane	354.04859	8.92	1.26	2.94	1.05E-11
scyllo-Inositol	365.1554	19.73	1.08	0.48	1.01E-08
Stearic acid	116.0083	20.24	1.01	0.40	1.11E-07
**Preoperative and postoperative serum**					
Stearic acid	298.2655	21.19	1.38	1.91	1.35E-02
3-Hydroxybutyric acid	86.0744	8.93	2.13	5.99	2.90E-02
9,12-Octadecadienoic acid	264.2632	20.98	2.24	3.63	6.28E-03
Cholestane-3,5-diol	329.3586	28.00	1.35	2.58	1.40E-02
Cholesterol	340.3024	20.98	2.16	3.50	4.01E-04
Glucose	96.0857	20.96	2.12	3.31	1.49E-02
elaidic acid	339.2998	20.97	2.16	3.17	8.60E-03
γ-Tocopherol	504.4245	27.86	1.58	2.35	8.40E-03
L-5-Oxoproline	258.0370	13.73	1.92	4.68	2.11E-03
Oleic acid	138.1276	20.96	2.19	3.51	1.31E-02
Palmitic acid	285.2381	19.40	1.44	2.04	1.60E-02
trans-13-Octadecenoic acid	84.0748	20.97	2.11	3.10	1.69E-02
Xanthotoxin	356.3373	21.18	1.06	1.78	1.33E-02

**Figure 6:**
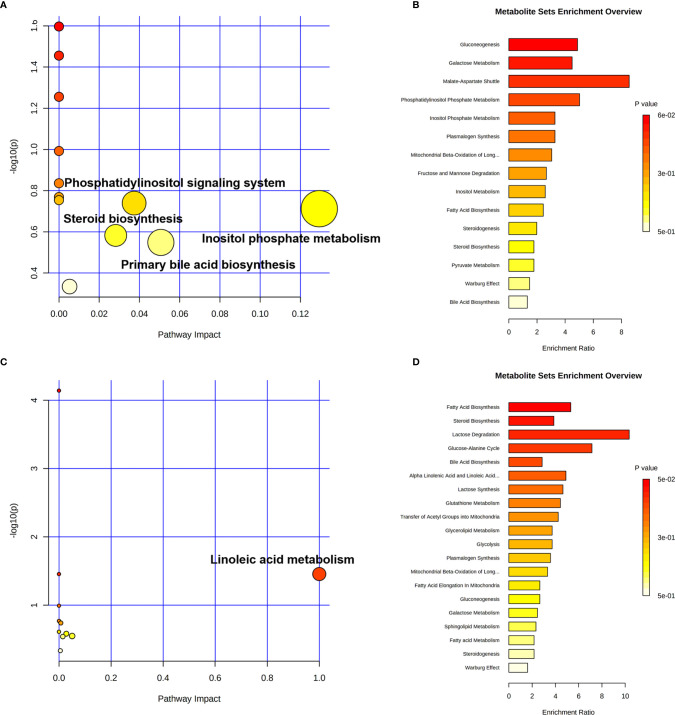
Pathway enrichment analysis of CRC in cancer tissue and paracarcinoma tissue samples **(A, B)** and preoperative and postoperative serum of 2-week sample **(C, D)** levels.

More detailed analyses of the most relevant pathways and networks for CRC were performed by MetaboAnalyst 5.0, which is a free web-based tool that combines results from powerful pathway enrichment analysis involved in the conditions under study. Metabolic pathway analysis revealed (**Table 3** and **Figure 6**) that 12 pathways contributed to CRC in tissue level, including inositol phosphate metabolism, primary bile acid biosynthesis, steroid biosynthesis, and phosphatidylinositol signaling system. At the same time, there are 12 pathways (**Table 3** and **Figure 6**) contributing to CRC in serum level, including linoleic acid metabolism, primary bile acid biosynthesis, and steroid biosynthesis. Ultimately, the most influential pathways contributing to CRC were inositol phosphate metabolism, primary bile acid biosynthesis, phosphatidylinositol signaling system, and linoleic acid metabolism.

**Table 3:** Result from ingenuity pathway analysis with MetaboAnalyst 5.0.

Pathway name	Match status	p	-log(p)	FDR	Impact
**Cancer tissue and paracarcinoma tissue**					
Biosynthesis of unsaturated fatty acids	2/36	0.025294	1.579	1	0
Synthesis and degradation of ketone bodies	1/5	0.035028	1.4556	1	0
Ascorbate and aldarate metabolism	1/8	0.055506	1.2557	1	0
Butanoate metabolism	1/15	0.10176	0.99242	1	0
Pyruvate metabolism	1/22	0.14595	0.83581	1	0
Glycolysis/gluconeogenesis	1/26	0.1703	0.76879	1	0
Galactose metabolism	1/27	0.17629	0.75378	1	0
Phosphatidylinositol signaling system	1/28	0.18224	0.73936	1	0.03736
Inositol phosphate metabolism	1/30	0.19402	0.71216	1	0.12939
Steroid biosynthesis	1/42	0.26153	0.58248	1	0.0282
Primary bile acid biosynthesis	1/46	0.28286	0.54843	1	0.05065
Steroid hormone biosynthesis	1/85	0.46338	0.33406	1	0.00528
**Preoperative and postoperative serum**					
Biosynthesis of unsaturated fatty acids	4/36	7.2193E-05	4.1415	0.0060643	0
Synthesis and degradation of ketone bodies	1/5	0.035028	1.4556	0.98079	0
Linoleic acid metabolism	1/5	0.035028	1.4556	0.98079	1
Butanoate metabolism	1/15	0.10176	0.99242	1	0
Glycolysis/gluconeogenesis	1/26	0.1703	0.76879	1	2.10E-04
Glutathione metabolism	1/28	0.18224	0.73936	1	0.00709
Fatty acid elongation	1/39	0.24515	0.61057	1	0
Fatty acid degradation	1/39	0.24515	0.61057	1	0
Steroid biosynthesis	1/42	0.26153	0.58248	1	0.0282
Primary bile acid biosynthesis	1/46	0.28286	0.54843	1	0.05065
Fatty acid biosynthesis	1/47	0.2881	0.54045	1	0.01473
Steroid hormone biosynthesis	1/85	0.46338	0.33406	1	0.00528
Biosynthesis of unsaturated fatty acids	4/36	7.2193E-05	4.1415	0.0060643	0
Synthesis and degradation of ketone bodies	1/5	0.035028	1.4556	0.98079	0
Linoleic acid metabolism	1/5	0.035028	1.4556	0.98079	1

### Signaling Networks

**Figure 7:**
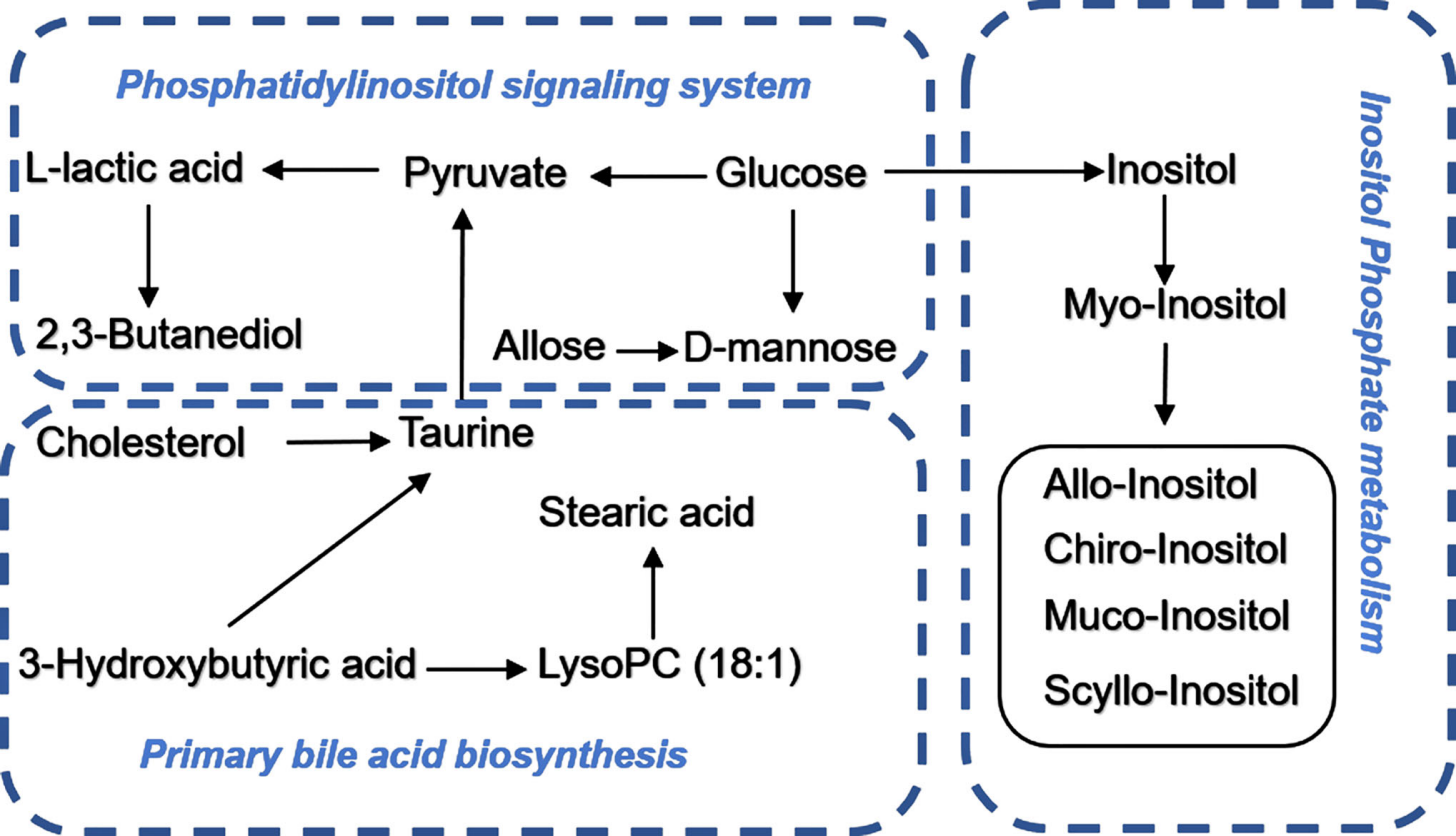
Schematic diagram of the disturbed metabolic pathway related to CRC.

Identified potential biomarkers were leading in the KEGG (http://www.kegg.jp/) in finding interactions, which show the relationships among these signal pathways. The networks were primarily related to inositol phosphate metabolism, primary bile acid biosynthesis, steroid biosynthesis, phosphatidylinositol signaling system, and linoleic acid metabolism. According to the pathway flow analysis, the primary bile acid biosynthesis pathway was deemed to be the upstream signaling network. Compared with the paracarcinoma tissue/preoperative serum, the levels of 2,3-butanediol, cholesterol, muco-inositol, oleic acid, and stearic acid were significantly downregulated. Conversely, L-lactic acid, allose, cholestane-3,5-diol, and glucose were markedly upregulated (**Figure 7**).

## Discussion

Colorectal cancer is the third leading cause of cancer-related deaths, and late-stage diagnosis is a major cause of morbidity and mortality of CRC, greatly threatening the health of humans (13, 14). The incidence of CRC has been rising continuously in recent decades, resulting in about 900,000 deaths per year globally [15-16]. There is thus an urgent need to discriminate accurate and non-invasive biomarkers to assist the early diagnosis and clinical management of CRC (15, 16). However, metabolomics is usually utilized to discriminate the potential biomarkers and, for the purpose of the study, the complexity and hugeness of metabolic networks based on a limited number of single pathways to characterize pathological states in animals and human (17).

In this study, the potential mechanisms of CRC, through gas chromatography–tandem mass spectrometry and multivariate statistics analysis, were assessed. A metabolic profiling method based on gas chromatography–tandem mass spectrometry coupled with multivariate statistical analysis including principal component analysis and orthogonal partial least square-discriminant analysis was employed to discriminate the groups, screen differential metabolites, identify the significant metabolites, and illustrate the mechanisms of disease. We gave an illustrative case to show that metabolomics is an innovative method for exploring disease biomarkers or intervention-related perturbed metabolic pathways (18).

In this study, untargeted GC-MS-based tissue (cancer tissue and paracarcinoma tissue) and serum (preoperative and postoperative of 2 weeks) metabolomics was applied to investigate the metabolic state of 50 human subjects. The itemization of CRC consensus molecular subtypes was performed in an effort to explicate the clinical heterogeneity through data analysis results (19). Additionally, an eight-biomarker panel (L-lactic acid, 2,3-butanediol, cholesterol, allose, malic acid, muco-inositol, oleic acid, stearic acid) can differentiate well between cancer tissue and paracarcinoma tissue. A six-biomarker (cholesterol, oleic acid, stearic acid, cholestane-3,5-diol, glucose, 3-hydroxybutyric acid) can differentiate well between the preoperative and postoperative sera of 2 weeks. As a consequence of the above, the levels of 2,3-butanediol, cholesterol, muco-inositol, oleic acid, and stearic acid were significantly upregulated. Conversely, L-lactic acid, allose, cholestane-3,5-diol, and glucose were markedly downregulated. CRC is closely related to metabolic dysfunction in the pathway of inositol phosphate metabolism, primary bile acid biosynthesis, steroid biosynthesis, phosphatidylinositol signaling system, and linoleic acid metabolism.

Glucose metabolism was utilized by cancer cells to provide sufficient metabolite precursors and energy to sustain fast cell growth (20, 21). Increased glucose uptake and enhanced glycolysis has been identified as a hallmark of cancer cells, with upregulated levels of transporters and enzymes involved in glucose metabolism. In our study, we found that glucose in preoperative CRC serum decreased obviously then that in 2-week postoperative serum. The decrease of glucose indicates that glycolysis is a metabolic alteration that appears early on the development of CRC to provide energy for cancer cell growth [22]. Inducing a condition of “hunger” in the cancer cells could be an effective method to improve the disease and possibly even treat the disease (22).

As the molecular skeleton of inositol hexaphosphate (IP6), a carbohydrate, and the precursor of phosphorylated compounds (23), inositol is primarily utilized to treat CRC (24) and other diseases. Inositol exhibits its biological activity on anticancer, and it synergistically reinforces the inhibitory pesticide effects of IP6 on the growth of colon and mammary cancers (24). It was shown that inositol decreased to about 42% in tumor tissues compared to non-tumor tissues. Simultaneously, with the reduction of inositol, an inhibitor of cancer, the cancer cell grows faster. Therefore, inositol might be a useful tool to inhibit the development and progression of CRC (25).

Although cholesterol is essential in our body, its levels are related with increased CRC risk and the treatment of statin could decrease CRC risk in older adults under 75 years of age (26). In cholesterol, for example, accumulation is observed in tumors from gastrointestinal cancer patients through increased low-density lipoprotein receptor (LDLR) and decreased ATP-binding cassette transporter (ABCA1) expression (27). Researchers interpreted that the CRC liver metastasis-specific cholesterol metabolic pathway is established for colonization of metastatic CRC cells (27). Simultaneously, inhibiting this CRC liver metastasis-specific cholesterol metabolic pathway could suppress CRC liver metastasis. Finally, it was confirmed that targeting the cholesterol biosynthesis pathway may be a promising therapy for liver metastasis of CRC (28). Cholesterol is an important metabolite participating in bile acid biosynthesis whose levels had increased in the CRC. Simultaneously, bile acids are cholesterol derivatives with detergent properties, are normally seen in the intestine, and have been suggested since 1939 as the tumor-promoting agents (29).

## Conclusion

Our research concentrated on the colorectal cancer patients based on untargeted GC-MS-based tissue (cancer tissue and paracarcinoma tissue) and serum (preoperative and postoperative of 2 weeks) metabolomics methods. Finally, 17 tissue and 13 serum candidate ions were selected based on their corresponding retention time, p-value, m/z, and VIP value. The results show that the trends of stearic acid and cholesterol are consistent, which were the most promising biomarkers. This also means that these metabolites might have important clinical significance for the detection of CRC. Metabolic pathway analysis revealed that 12 pathways contributed to CRC in tissue level, including inositol phosphate metabolism, primary bile acid biosynthesis, steroid biosynthesis, and phosphatidylinositol signaling system. At the same time, there are 12 pathways contributing to CRC in serum level, including linoleic acid metabolism, primary bile acid biosynthesis, and steroid biosynthesis. Ultimately, the most influential pathways contributing to CRC were inositol phosphate metabolism, primary bile acid biosynthesis, phosphatidylinositol signaling system, and linoleic acid metabolism. Further research will be conducted to determine if these biomarkers could be fully integrated into application for early diagnosis of CRC.

## Data Availability Statement

The original contributions presented in the study are included in the article/**Supplementary Material**. Further inquiries can be directed to the corresponding authors.

## Ethics Statement

The studies involving human participants were reviewed and approved by the Nanjing Hospital of Chinese Medicine Affiliated to Nanjing University of Chinese Medicine. The patients/participants provided their written informed consent to participate in this study.

## Author Contributions

GZ, DK, and ZF designed the research. WW, YW, and GZ performed the experiments. BP and FS collected the samples. GZ and WW analyzed the data. GZ, YW, and YZ wrote the manuscript. All authors contributed to the article and approved the submitted version.

## Funding

Supported by Nanjing Youth Talent Training Plan of TCM(ZYQ20006), Nanjing Medical Science and Technology Development Project (YKK20167), Training Plan of Clinical Medicine Research Center Jointly Built by Ministry and Province in Nanjing (GCYJZX-2019), and Nanjing Chinese Medicine Modernization and Big Data Research Center, Gu Xiaosong’s Academician Workstation.

## Conflict of Interest

The authors declare that the research was conducted in the absence of any commercial or financial relationships that could be construed as a potential conflict of interest.

## Publisher’s Note

All claims expressed in this article are solely those of the authors and do not necessarily represent those of their affiliated organizations, or those of the publisher, the editors and the reviewers. Any product that may be evaluated in this article, or claim that may be made by its manufacturer, is not guaranteed or endorsed by the publisher.
