# Supplementary material for: Untargeted GC-MS-Based Metabolomics for Early Detection of Colorectal Cancer
Source: Front Oncol. 2021 Nov 4;11:729512. doi: 10.3389/fonc.2021.729512 (PMC8599589; doi:10.3389/fonc.2021.729512)
Supplement: Supplementary file 1 [file Image_1.pdf]

Supplementary Material

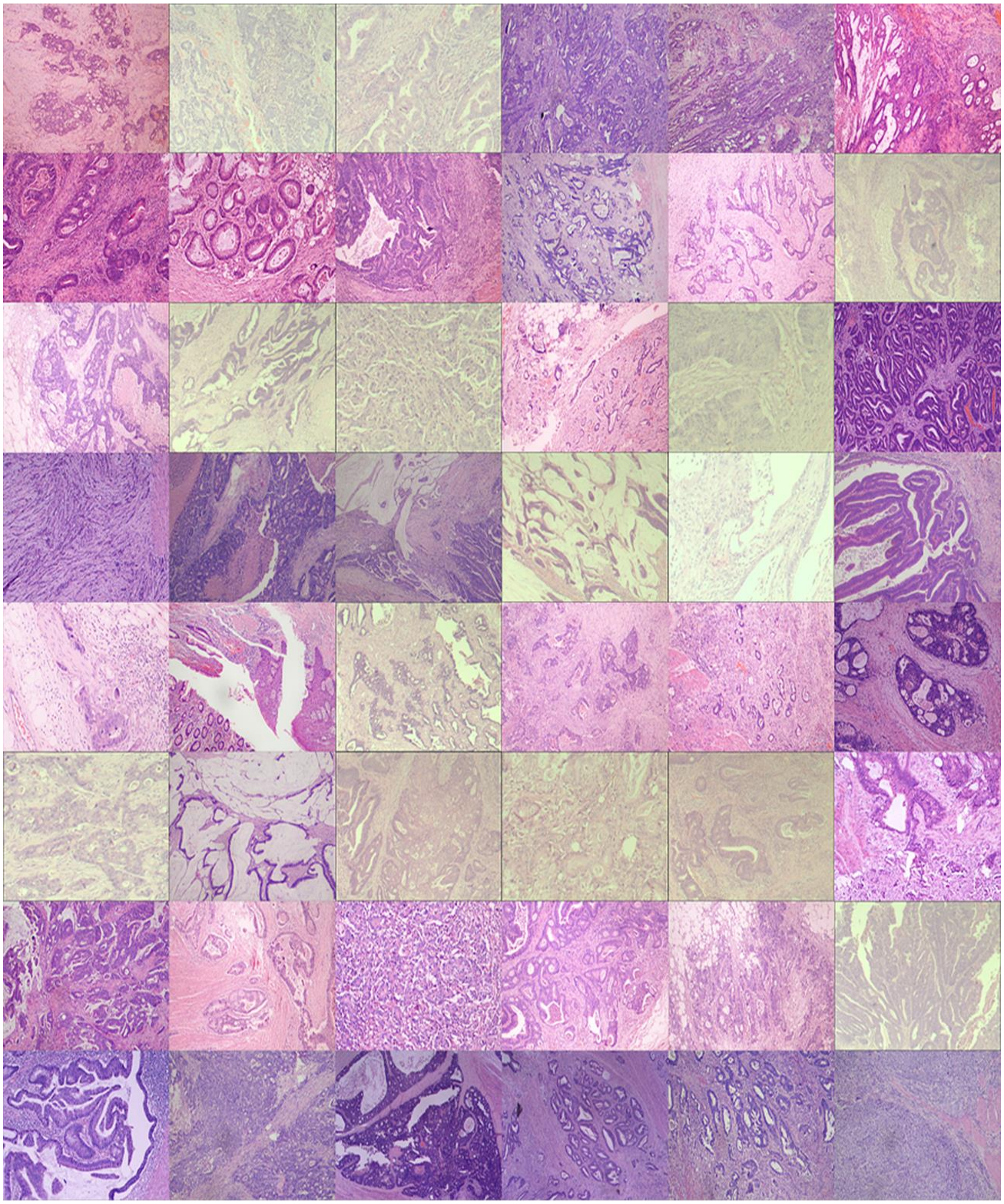

Figure S1 Histopathological section photographs of CRC patients for H&E analysis (100× magnifification). (Place in the order of Table S1 from left to right)

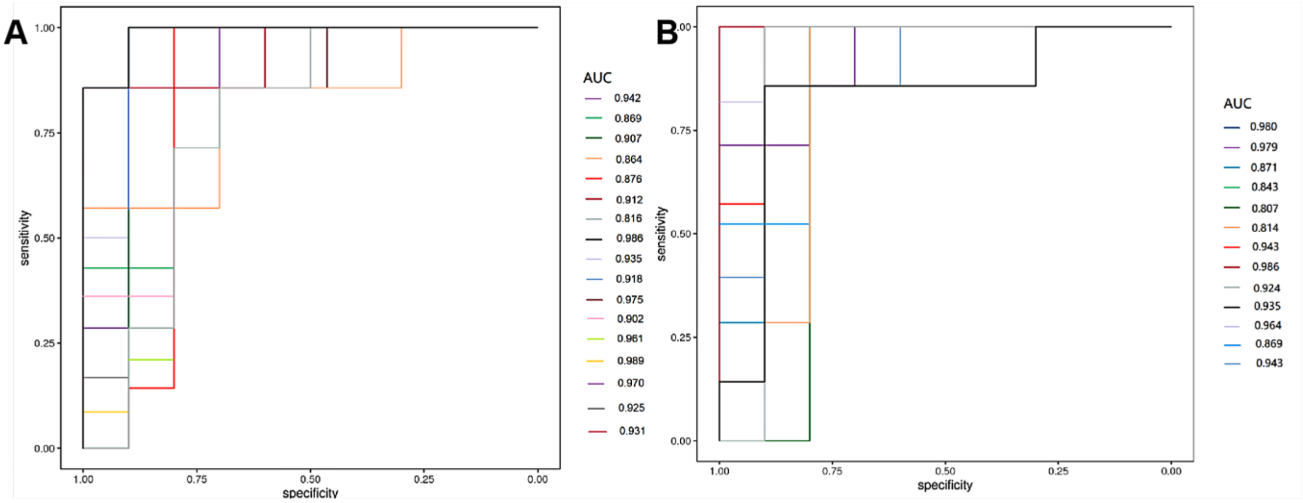

Figure S2 The ROC curve of potential biomarkers among the tissue (A) and serum (B).
