# Supplementary material for: Untargeted GC-MS-Based Metabolomics for Early Detection of Colorectal Cancer
Source: Front Oncol. 2021 Nov 4;11:729512. doi: 10.3389/fonc.2021.729512 (PMC8599589; doi:10.3389/fonc.2021.729512)
Supplement: Supplementary file 2 [file Table_1.docx]

Supplementary Material

Table S1 Clinical features of colorectal cancer patients enrolled in this study.

| Gender  Male/Female | Age | Blood glucose  （mmol/L） | Height  (m) | Weight  (Kg) | BMI  (kg/m^2^) | Pathologic materials | | | | | | | |
| --- | --- | --- | --- | --- | --- | --- | --- | --- | --- | --- | --- | --- | --- |
|  |  |  |  |  |  | Tumor sites | Pathologic subtypes | Tumor size(cm) | Differentiation | Infiltration | Lymphatic metastasis | Distant metastasis | Tumor stage |
| Male | 58 | 5.01 | 1.65 | 65.00 | 23.88 | Colorectal | Adenocarcinoma | 4×3.5×1 | Medium | Adventitia | 4/16 | N/A | Ⅲ |
| Male | 85 | 5.41 | 1.64 | 66.00 | 24.54 | Colorectal | Adenocarcinoma | 5.7×5×2.5 | Medium | Deep myometrial | 0/29 | N/A | Ⅰ |
| Male | 62 | 4.78 | 1.75 | 70.00 | 22.86 | Sigmoid colon | Adenocarcinoma | 6.5×4 | Medium | Serosal | 1/25 | N/A | Ⅲ |
| Female | 48 | 4.83 | 1.58 | 51.00 | 20.43 | Colorectal | Adenocarcinoma | 3×2.4×0.8 | Medium | Submucosal | 0/16 | N/A | Ⅰ |
| Female | 71 | 4.92 | 1.68 | 70.00 | 24.80 | Colorectal | Adenocarcinoma | 5.8×3.5 | Medium-Low | Serosal | 1/22 | N/A | Ⅲ |
| Female | 36 | 4.99 | 1.65 | 70.00 | 25.71 | Colorectal | Adenocarcinoma | 6×4 | Medium | Adventitia | 0/18 | N/A | Ⅱ |
| Male | 65 | 4.91 | 1.75 | 63.00 | 20.57 | Descending colon | Adenocarcinoma | 2.5×2×0.8 | Medium | Serosal | 0/12 | N/A | Ⅱ |
| Male | 54 | 4.92 | 1.73 | 71.00 | 23.72 | Ascending colon | Adenocarcinoma | 8×6.5×2 | Medium | Peripheral adipose | 0/21 | N/A | Ⅱ |
| Male | 80 | 5.58 | 1.60 | 60.00 | 23.44 | Colorectal | Adenocarcinoma | 4.5×4.5×0.6 | Medium | Deep myometrial | 0/10 | N/A | Ⅰ |
| Female | 67 | 6.62 | 1.63 | 65.00 | 24.46 | Sigmoid colon | Adenocarcinoma | 4×3×0.4 | Medium | Serosal | 8/21 | N/A | Ⅲ |
| Female | 85 | 4.69 | 1.56 | 70.00 | 28.76 | Colorectal | Adenocarcinoma | 3×2 | Medium | Adventitia | 0/17 | N/A | Ⅱ |
| Male | 62 | 5.91 | 1.71 | 62.00 | 21.20 | Ascending colon | Adenocarcinoma | 5.2×3 | Medium-Low | Serosal | 0/36 | N/A | Ⅱ |
| Male | 78 | 4.92 | 1.60 | 58.50 | 22.85 | Colorectal | Adenocarcinoma | 5×4 | Medium | Serosal | 9/19 | N/A | Ⅲ |
| Female | 67 | 5.11 | 1.52 | 47.00 | 20.34 | Ascending colon | Adenocarcinoma | 4.3×3.7 | Medium | Serosal | 0/30 | N/A | Ⅱ |
| Female | 84 | 4.88 | 1.54 | 50.00 | 21.08 | Ascending colon | Adenocarcinoma | 11×7×3 | Low | Submucosal | 14/24 | N/A | Ⅲ |
| Female | 66 | 4.11 | 1.60 | 45.00 | 17.58 | Sigmoid colon | Adenocarcinoma | 3.5×2.5 | Medium | Submucosal | 5/22 | N/A | Ⅲ |
| Male | 72 | 5.72 | 1.67 | 75.00 | 26.89 | Ascending colon | Adenocarcinoma | 4×3 | Medium | Serosal | 0/17 | N/A | Ⅱ |
| Male | 44 | 4.08 | 1.69 | 52.90 | 18.52 | Colorectal | Adenocarcinoma | 4×4 | Medium | Adventitia | 1/16 | N/A | Ⅲ |
| Male | 43 | 4.57 | 1.77 | 75.00 | 23.94 | Ascending colon | Adenocarcinoma | 5×4×3 | Low | Serosal | 10/17 | N/A | Ⅲ |
| Male | 66 | 4.23 | 1.75 | 85.00 | 27.76 | Colorectal | Adenocarcinoma | 2.5×2.2×1 | Medium-Low | Deep myometrial | 0/10 | N/A | Ⅰ |
| Female | 61 | 4.77 | 1.60 | 45.00 | 17.58 | Ascending colon | Adenocarcinoma | 6×5×1.4 | Medium | Serosal | 9/17 | N/A | Ⅲ |
| Male | 46 | 5.11 | 1.70 | 77.00 | 26.64 | Ascending colon | Adenocarcinoma | 5.3×4.2×1.5 | Medium-Low | Serosal | 0/20 | N/A | Ⅱ |
| Female | 57 | 5.27 | 1.60 | 58.00 | 22.66 | Sigmoid colon | Mucoid carcinoma | 5×4 | Low | Subserosal | 0/16 | N/A | Ⅲ |
| Male | 67 | 4.81 | 1.60 | 77.80 | 30.39 | Ascending colon | Adenocarcinoma | 4×3×2.2 | High | Submucosal | 0/18 | N/A | Ⅰ |
| Male | 84 | 5.97 | 1.70 | 70.00 | 24.22 | Colorectal | Adenocarcinoma | 6×5 | Medium-Low | Adventitia | 0/13 | N/A | Ⅱ |
| Male | 75 | 4.56 | 1.70 | 65.00 | 22.49 | Colorectal | Adenocarcinoma | 4×3.2×0.5 | Medium | Adventitia | 0/20 | N/A | Ⅱ |
| Female | 50 | 4.39 | 1.62 | 50.00 | 19.05 | Ascending colon | Adenocarcinoma | 6×5 | Medium | Serosal | 2/23 | lung | Ⅳ |
| Male | 74 | 4.34 | 1.70 | 75.00 | 25.95 | Colorectal | Adenocarcinoma | 3.5×3×0.8 | Medium | Adventitia | 1/24 | N/A | Ⅲ |
| Female | 70 | 5.40 | 1.50 | 39.00 | 17.33 | Colorectal | Adenocarcinoma | 4×3.5 | Medium | Adventitia | 0/23 | N/A | Ⅱ |
| Male | 69 | 6.08 | 1.73 | 65.00 | 21.72 | Sigmoid colon | Adenocarcinoma | 8×5.5×5 | Medium | Serosal | 0/33 | N/A | Ⅱ |
| Female | 68 | 4.88 | 1.60 | 59.00 | 23.05 | Colorectal | Adenocarcinoma | 5.5×4.2 | Low | Subserosal | 7/19 | N/A | Ⅲ |
| Female | 72 | 6.10 | 1.63 | 58.00 | 21.83 | Descending colon | Mucoid carcinoma | 6.5×4×1.5 | Low | Subserosal | 2/17 | N/A | Ⅲ |
| Female | 64 | 6.80 | 1.55 | 50.00 | 20.81 | Colorectal | Adenocarcinoma | 3.5×2.2 | Medium | Deep myometrial | 2/29 | N/A | Ⅲ |
| Male | 71 | 5.30 | 1.65 | 78.00 | 28.65 | Ascending colon | Adenocarcinoma | 7.5×6 | Medium-Low | Serosal | 0/28 | N/A | Ⅲ |
| Female | 70 | 5.53 | 1.63 | 69.50 | 26.16 | Sigmoid colon | Adenocarcinoma | 6.8×4.7×3.6 | Medium | Serosal | 0/20 | N/A | Ⅱ |
| Male | 55 | 5.01 | 1.68 | 60.00 | 21.26 | Sigmoid colon | Adenocarcinoma | 4.5×3.5 | Medium | Subserosal | 0/18 | N/A | Ⅱ |
| Female | 73 | 4.62 | 1.58 | 47.50 | 19.03 | Ascending colon | Adenocarcinoma | 4×3.2×1 | Medium-Low | Serosal | 0/22 | N/A | Ⅱ |
| Male | 45 | 5.22 | 1.76 | 75.00 | 24.21 | Ascending colon | Adenocarcinoma | 5×3.5 | Medium | Serosal | 2/20 | N/A | Ⅲ |
| Female | 85 | 5.05 | 1.60 | 44.00 | 17.19 | Ascending colon | Adenocarcinoma | 6×4.5×0.5 | Medium-Low | Serosal | 0/20 | N/A | Ⅱ |
| Male | 69 | 4.28 | 1.70 | 75.00 | 25.95 | Colorectal | Adenocarcinoma | 3×2×1 | Medium | Submucosal | 0/12 | N/A | Ⅰ |
| Female | 53 | 5.24 | 1.53 | 51.00 | 21.79 | Colorectal | Adenocarcinoma | 4×3 | Medium | Subserosal | 8/16 | N/A | Ⅲ |
| Male | 77 | 5.66 | 1.70 | 62.90 | 21.76 | Sigmoid colon | Adenocarcinoma | 9×6×3 | Medium | Serosal | 0/26 | N/A | Ⅱ |
| Male | 64 | 5.06 | 1.75 | 65.00 | 21.22 | Sigmoid colon | Adenocarcinoma | 7×4×3.5 | Medium | Submucosal | 0/8 | N/A | Ⅰ |
| Female | 45 | 4.45 | 1.60 | 56.00 | 21.88 | Sigmoid colon | Adenocarcinoma | 9×8×2 | Medium-Low | Subserosal | 1/25 | N/A | Ⅲ |
| Male | 71 | 5.79 | 1.72 | 69.00 | 23.32 | Colorectal | Adenocarcinoma | 5×3 | Medium-Low | Adventitia | 3/22 | N/A | Ⅲ |
| Male | 63 | 3.90 | 1.70 | 70.00 | 24.22 | Ascending colon | Adenocarcinoma | 3.2×3 | Medium | Serosal | 0/35 | N/A | Ⅱ |
| Male | 78 | 4.49 | 1.78 | 60.00 | 18.94 | Colorectal | Adenocarcinoma | 5×3×0.8 | Medium | Adventitia | 0/19 | N/A | Ⅱ |
| Male | 62 | 5.19 | 1.70 | 83.00 | 28.72 | Colorectal | Adenocarcinoma | 6.5×5.2×0.8 | Low | Adventitia | 3/16 | N/A | Ⅲ |
